# Intersecting health burdens: oral health, non-communicable disease screening, and women's health in rural Tanzania

**DOI:** 10.3389/froh.2025.1644013

**Published:** 2025-09-17

**Authors:** Priyanka Gudsoorkar, Anay Dudhbhate, Jessica Klabak, Steve Klabak, Rachael Nolan

**Affiliations:** 1 Department of Environmental & Public Health Sciences, University of Cincinnati, Cincinnati, OH, United States; 2 Solidarity Dental Foundation, Cincinnati, OH, United States; 3 Lumescent Global Health, Fraser, CO, United States

**Keywords:** oral-general health, oral health, global oral health, oral health-related quality of life (OHRQOL), women's health

## Abstract

**Introduction:**

This cross-sectional study explored the intersection of oral health, other non-communicable diseases (NCDs), and women's health in rural Tanzania, using community-based screenings to identify syndemic patterns of vulnerability and inform integrated care strategies.

**Methods:**

A total of 224 adult women were recruited during outreach events in three Rorya District villages of Burere, Nyambogo, and Roche in July 2023. Clinical oral examinations were conducted alongside biomarker analysis using the PerioMonitor™, as well as survey-based assessments, including the Oral Health–Related Quality of Life (OHRQoL) scale and the Hologic Global Women's Health Index (HGWI). A subsample of 45 participants underwent additional screening for blood pressure (BP) and blood glucose levels.

**Results:**

Only 18.2% of participants reported having received prior BP screening. The mean DMFT score was 5.16, and 40% of the sample showed elevated periodontal inflammation. The average OHRQoL score was 11.15, indicating substantial functional and psychosocial impacts. Among those screened further, 49% were hypertensive, 2% were hyperglycemic, and 18% were hypoglycemic, most without a prior diagnosis.

**Conclusions:**

Community-based screening proved both feasible and impactful, uncovering overlapping burdens of untreated oral disease, metabolic dysregulation, and unmet preventive care. These findings reflect the structural and clinical dimensions of oral health inequity and align with syndemic theory, underscoring the need for integrated, gender-responsive, and culturally grounded interventions. They also offer a foundation for scalable, sustainable models of care in low-resource settings.

## Introduction

Non-communicable diseases (NCDs) are a growing public health challenge in Tanzania, where they account for nearly one-third of all deaths (1, 2). This burden is especially pronounced in rural districts like Rorya, where health infrastructure is limited, preventative services are scarce, and communities face significant barriers to early detection and treatment (3). These conditions not only affect daily functioning and well-being but also place considerable strain on already under-resourced health systems (1).

Oral diseases, particularly dental caries and periodontal inflammation, are among the most common and neglected NCDs in rural Tanzania (4). Despite their systemic health implications, they remain largely absent from national health strategies. High rates of untreated oral disease stem from traditional hygiene practices, limited access to care, and behaviors like toothbrush sharing (5–9). At the same time, the rising prevalence of hypertension and diabetes across sub-Saharan Africa has outpaced rural screening capacity, leaving many cases undiagnosed (10, 11). These overlapping burdens underscore the urgency of integrated care models that address both oral and systemic health (12).

Women's health is also inadequately addressed in these settings. Progress toward maternal health targets, including Sustainable Development Goal 3.1, remains limited (13). Inconsistent data and persistent systemic barriers, such as workforce shortages, fragmented services, and financial or geographic constraints, continue to impede access to even basic preventative care (14, 15). These challenges are especially acute in rural areas, where women often depend on under-resourced primary care systems that are ill-equipped to meet their broader health needs. As a result, preventable conditions related to reproductive health, chronic disease, and mental well-being frequently go undetected and untreated.

In response, this study applies a syndemic framework to examine the interconnected burdens of oral disease, metabolic risk, and gender-based disparities in rural Tanzania. Syndemic theory underscores how co-occurring health conditions, shaped by structural and social inequities, interact to intensify vulnerability in marginalized populations. Drawing on three years of community-based research in the Rorya District, we integrate clinical dental assessments, biomarker screening, and validated survey tools to generate localized, multidimensional data. We aim to inform culturally responsive, equity-oriented interventions and support more inclusive health planning in underserved settings.

## Background

Oral health is essential to overall well-being, enabling daily functions such as eating, speaking, and socializing without pain (16–20). In rural Tanzania, however, it remains a significant concern due to limited access to hygiene tools and continued reliance on traditional practices like chewing sticks (21). Dental caries and periodontal disease are widespread, contributing to chronic pain, inflammation, and reduced quality of life (22). Although preventive measures such as brushing with fluoridated toothpaste are effective, they remain inaccessible for many due to economic and logistical barriers (23).

To assess oral health status, this study builds upon previous qualitative research (22) and employs PerioMonitor™, a rapid, point-of-care, non-invasive tool that measures neutrophil activity in oral rinse to detect subclinical periodontal inflammation (24). Its portability and minimal equipment needs make it well-suited for use in low-resource, field-based settings. We also conduct clinical examinations using the Decayed, Missing, and Filled Teeth (DMFT) index (25), and evaluate the self-reported functional and psychosocial impacts through the Oral Health-Related Quality of Life (OHRQoL) (26) survey. Together, these tools provide a multidimensional picture of the oral health burden in this rural population.

In addition to oral health and biomarker assessments, this study includes screening for hypertension and diabetes, two common yet frequently undiagnosed NCDs in sub-Saharan Africa (24). Early detection is critical for timely education and intervention, especially in rural areas with limited diagnostic services. To further contextualize these clinical findings within the lived experiences of women, we administer the preventive care domain of the Hologic Global Women's Index (HGWI) (27), a tool used in over 140 countries to assess access to care, emotional well-being, safety, and basic needs. This multidimensional tool provides critical insight into the structural and social determinants shaping women's health in the Rorya District.

## Methods

### Study design

This cross-sectional study was conducted in July 2023 across the three rural villages of Burere, Nyambogo, and Roche in the Rorya District of Tanzania. Data collection was conducted in collaboration with the Village Life Outreach Project (VLOP) and local Tanzanian health partners, including the Roche Health Center and SHED (Supporting Health and Education in the District) sites. Ethical approval was obtained from the Institutional Review Board at the University of Cincinnati (Protocol No. 2022-0332) and the review board of SHED.

### Ethical considerations

Particular care was taken to address ethical and cultural considerations, especially given the possibility of uncovering undiagnosed conditions such as hypertension or abnormal glucose levels. When such findings occurred, participants were respectfully informed and referred to the nearest health facility in accordance with local protocols. Informed consent procedures were adapted to the local context to ensure clarity and comprehension in a low-literacy setting. Consent was obtained both verbally and in writing, with information presented in participants' preferred language (Swahili or English) using plain language and visual aids as needed. Trained bilingual researchers facilitated the consent process, ensuring that participants had ample opportunity to ask questions. These procedures reflect a commitment to ethical and reflexive research conduct that prioritizes participant well-being, autonomy, and dignity in resource-limited environments.

### Sampling and recruitment

A total of *n* = 224 adult women were recruited through purposive sampling at community health outreach events. Eligible participants were women aged 18 or older, resided in one of the study villages, and were able to communicate in Swahili or English. Each participant was assigned a unique, anonymized six-digit code to ensure confidentiality. Due to field constraints, including time, resources, and participant availability at mobile clinic sites, a subsample of 45 participants underwent additional screening for NCDs, including blood pressure (BP) and fasting glucose assessments. Participants received oral hygiene kits containing toothbrushes, toothpaste, and educational materials aligned with American Dental Association guidelines (28).

### Measures

Oral health was assessed through clinical examinations and self-reported surveys, while PerioMonitor™ data quantified neutrophil levels in oral rinse as a biomarker of subclinical periodontal inflammation. Moderate-to-high inflammation was defined by a PerioMonitor™ score ≥2. Neutrophil activity was visually interpreted based on a colorimetric shift on the test strip, ranging from light pink (low activity) to deep purple (high activity), corresponding to established markers of periodontal disease. Clinical examinations were conducted by calibrated examiners using a mouth mirror and dental probe under natural or artificial lighting.

Oral disease burden was measured using the DMFT Index, where a score ≥5 indicates poor oral health (29). Poor oral health–related quality of life was defined as an OHRQoL score ≥10, indicating notable functional, psychological, and social impacts associated with oral disease (30). To assess recent engagement with health services in the past year, all participants completed the preventive care domain of the HGWI. These instruments were orally administered in Swahili by bilingual researchers trained in culturally sensitive practices, with responses recorded on paper-based forms.

BP was measured following a five-minute seated rest using a sphygmomanometer and stethoscope, with hypertension defined according to American Heart Association guidelines (≥130/80 mmHg or current antihypertensive use) (31). Fasting capillary blood glucose was collected via finger prick and measured using a flash glucose monitor. Readings were classified using American Diabetes Association diagnostic thresholds of normal (70–100 mg/dl), hyperglycemic (≥126 mg/dl), or hypoglycemic (<70 mg/dl) (32).

### Data analysis and management

All data were de-identified and securely managed to protect participant confidentiality. Paper-based survey responses and clinical data were manually entered into Microsoft Excel (33) by the research team. Each entry was independently checked for accuracy by the lead researcher to ensure data integrity before analysis. Electronic files were stored on password-protected computers and backed up on encrypted drives accessible only to authorized study personnel.

Descriptive statistics were used to summarize participant demographics, clinical indicators, and survey responses. Frequencies and proportions were calculated for categorical variables, while means and standard deviations were computed for continuous variables, including DMFT indices, BP, blood glucose levels, and OHRQoL scores. Inferential analyses, including *t*-tests, were conducted to explore associations among oral health, NCDs, and women's health domains. Cumulative risk burden was assessed by identifying participants who met two or more of the following clinical criteria: elevated DMFT score (≥5), moderate-to-high PerioMonitor™ score (≥2), and evidence of metabolic dysregulation defined as either abnormal blood glucose levels (≥126 mg/dl or <70 mg/dl) or elevated BP (≥130/80 mmHg or current antihypertensive use). Participants meeting these thresholds were categorized into a high-risk group for subsequent comparisons. All analyses were performed in Python v3.12 using NumPy v2.1.3, Pandas v2.2.3, Seaborn v0.13.2, and Matplotlib v3.10.0. libraries (34) within the JupyterLab development environment (35).

## Results

### Descriptives

A total of (*n* = 224) female participants, with a mean age of 43.75 years (*SD* ± 17.62), were enrolled across the three rural Tanzanian study sites of Burere (*n* = 51), Nyambogo (*n* = 95), and Roche (*n* = 78) (see Table 1). Educational attainment was low among participants. Approximately 52% (*n* = 117) had only primary education, 32% (*n* = 72) had completed secondary or higher education, and 16% (*n* = 35) reported no formal education. Clinical examinations and oral health assessments were completed by 82% of participants (*n* = 183), while a subsample of 20% (*n* = 45) underwent BP and glucose screenings. The HGWI was administered to all participants (*n* = 224).

**Table 1:** Summary of health findings in the study sample by village, age, and income.

Health findings		Age		Systolic blood pressure (≥130 mmHg)		Diastolic blood pressure (≥80 mmHg)		Blood sugar (≥126 mg/dl or <70 mg/dl)		DMFT^a^ (≥5)		OHRQoL^b^ (≥10)	
		*n*	Mean	*n*	Mean	*n*	Mean	*n*	Mean	*n*	Mean	*n*	Mean
	Total	224	43.75	45	128.44	45	78.16	45	85.09	183	5.16	183	11.15
Village	Burere	51	41.92	20	130.25	20	78.00	20	83.40	51	5.88	51	11.00
	Nyambogo	95	50.11	2	127.50	2	75.00	2	94.00	54	5.07	54	13.57
	Roche	78	37.22	23	126.96	23	78.57	23	85.78	78	4.76	78	9.58
Age	15–50	141	32.17	27	123.15	27	76.93	27	83.74	117	5.11	117	10.28
	51–75	71	60.66	16	131.88	16	79.38	16	86.50	61	5.08	61	12.59
	76+	12	79.83	2	172.50	2	85.00	2	92.00	5	7.40	5	14.00
Income	Lowest 20%	43	40.35	11	125.45	11	79.09	11	79.91	35	3.77	35	10.60
	Second 20%	45	44.36	10	125	10	74.00	10	86.20	38	5.50	38	11.71
	Middle 20%	17	49.65	2	142.50	2	70.00	2	59.50	14	5.93	14	11.71
	Fourth 20%	38	45.39	6	133.33	6	81.67	6	74.33	30	5.40	30	11.10
	Highest 20%	29	47.14	6	124.17	6	78.33	6	86.00	23	4.39	23	11.74
	Unknown	52	41.04	10	132.00	10	80.70	10	100.7	43	6.00	43	10.65

a Decayed, missing and filled teeth.

b Oral health-related quality of life.

### Oral health burden

**Figure 1:**
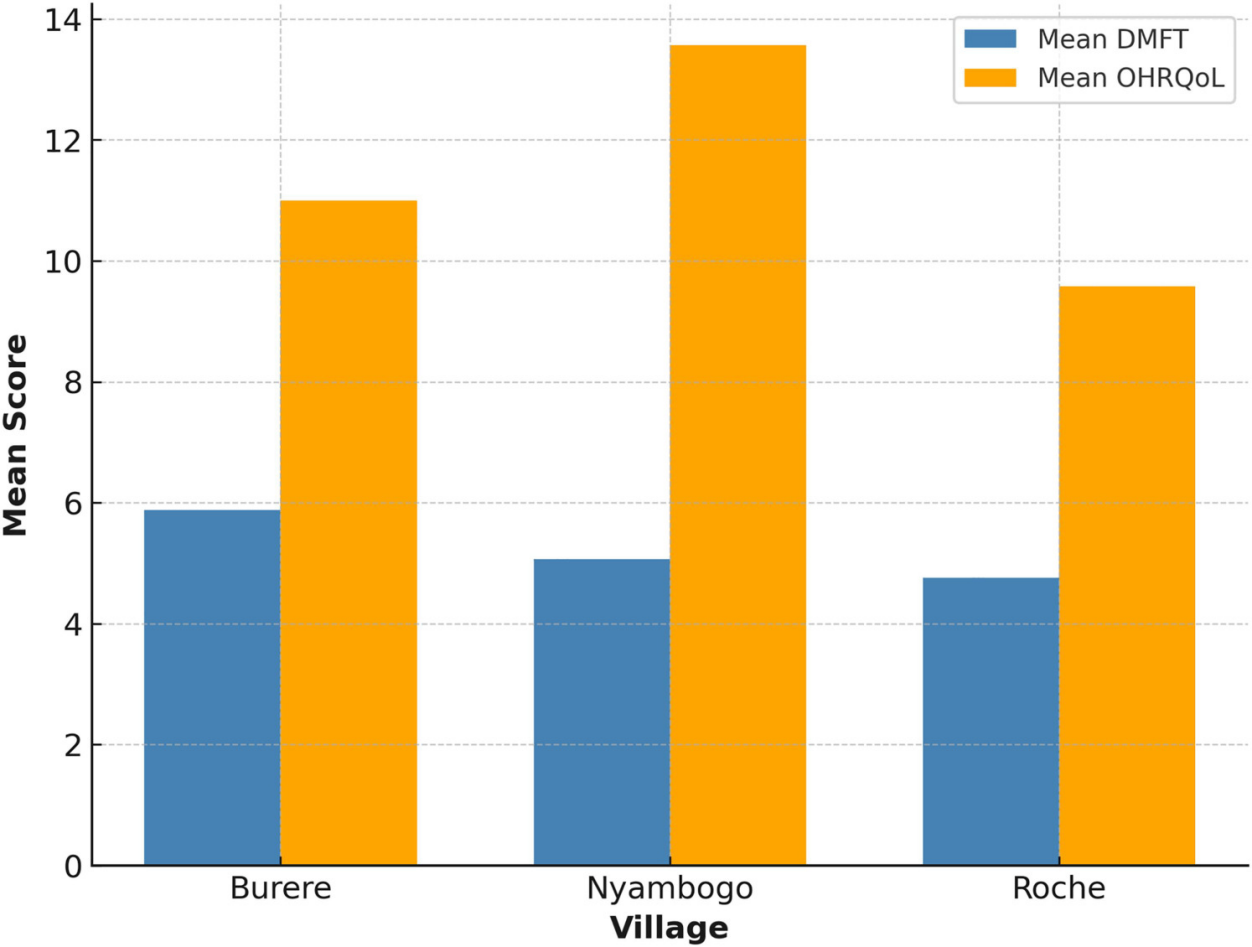
Mean DMFT and OHRQoL by village.

The mean DMFT score among all participants was 5.16 (*SD* ± 4.56), with decayed teeth comprising the most significant component (*x¯* = 3.4) (see Table 1). The average number of missing teeth was 1.75, and the number of filled teeth was rare (*x¯* = 0.03), reflecting limited access to dental care. DMFT scores varied across village sites (see Figure 1), with the highest average observed in Burere (*x¯* = 5.88), followed by Nyambogo (*x¯* = 5.07) and Roche (*x¯* = 4.76), but were not statistically significant (*p* > 0.05). Similarly, a one-way ANOVA revealed no significant differences in DMFT scores across select demographics [*F*(2,180) = 1.39, *p* > 0.05, *η*^2^ = 0.015].

OHRQoL assessments yielded a mean score of 11.15 and a median of 12 (*SD* ± 4.62), indicating poor oral health–related quality of life across the sample (see Table 1). Village-level averages revealed the highest scores in Nyambogo (*x¯* = 13.57), followed by Burere (*x¯* = 11.00), and the lowest in Roche (*x¯* = 9.58) (see Figure 1). Based on item-level responses, the most affected quality of life domains were physical pain (e.g., toothache, discomfort while eating) and psychological discomfort (e.g., self-consciousness, embarrassment). Both parametric ANOVA [*F*(2,180) = 0.19, *p* > 0.05, *η*^2^ = 0.002] and non-parametric Kruskal–Wallis testing (*H* = 1.85, *p* > 0.05) tests were employed; however, no statistically significant differences were found in OHRQoL scores across select demographics.

PerioMonitor™ assessments were conducted with 223 participants. Of the participants assessed, 40% demonstrated high neutrophil activity (*n* = 89; score ≥3), 29% showed moderate activity (*n* = 65; score ≥2 but <3), and 27% had low activity (*n* = 61; score <2), while 3% (*n* = 8) were disqualified due to incomplete or invalid samples. Visual interpretation of the test strip's colorimetric shift, from light pink to deep purple, reflected neutrophil activity consistent with established markers of periodontal disease.

### BP and glucose

Of the participants (*n* = 45) who underwent blood pressure screening, 49% (*n* = 22) were classified as hypertensive (see Table 1). Among those found to be hypertensive, 77% (*n* = 17) reported no prior diagnosis. The mean systolic BP was 128 mmHg (*SD* ± 19.48) and the mean diastolic was 78 mmHg (*SD* ± 10.36). Average BP readings across the three villages of Burere (*x¯* = 130/78 mmHg), Nyambogo (*x¯* = 128/75 mmHg), and Roche (*x¯* = 127/79 mmHg) were similar.

When stratified by cumulative risk, women in the high-risk group had a mean systolic BP of 134 mmHg and a mean DMFT score of 6.4. In contrast, those in the lower-risk group had a mean systolic pressure of 122 mmHg and a mean DMFT of 4.7. Welch's *t*-tests were conducted to compare both DMFT and OHRQoL scores between participants with and without hypertension. Results indicated that DMFT (*t* = −0.23, *p* = 0.82) and OHRQoL scores (*t* = −0.14, *p* = 0.89) were not significantly associated with hypertension status.

Among the 45 participants screened for diabetes, 80% (*n* = 36) were normoglycemic (70–99 mg/dl), 18% (*n* = 8) were hypoglycemic (<70 mg/dl), and 2% (*n* = 1) were hyperglycemic (≥126 mg/dl). The mean fasting glucose level was 85 mg/dl (*SD* ± 20.89). Average fasting glucose levels across the three villages were highest in Nyambogo (*x¯* = 94 mg/dl), followed by Roche (*x¯* = 86 mg/dl) and Burere (*x¯* = 83 mg/dl). An ANOVA was conducted to compare DMFT scores across blood glucose categories; however, the analysis was restricted to participants classified as hypoglycemic and normoglycemic due to only one participant meeting criteria for hyperglycemia. The analysis revealed no statistically significant difference between groups (*F*₁,₄₂ = 0.21, *p* = 0.65), suggesting that blood glucose did not significantly influence DMFT scores in this sample.

### Women's health

**Figure 2:**
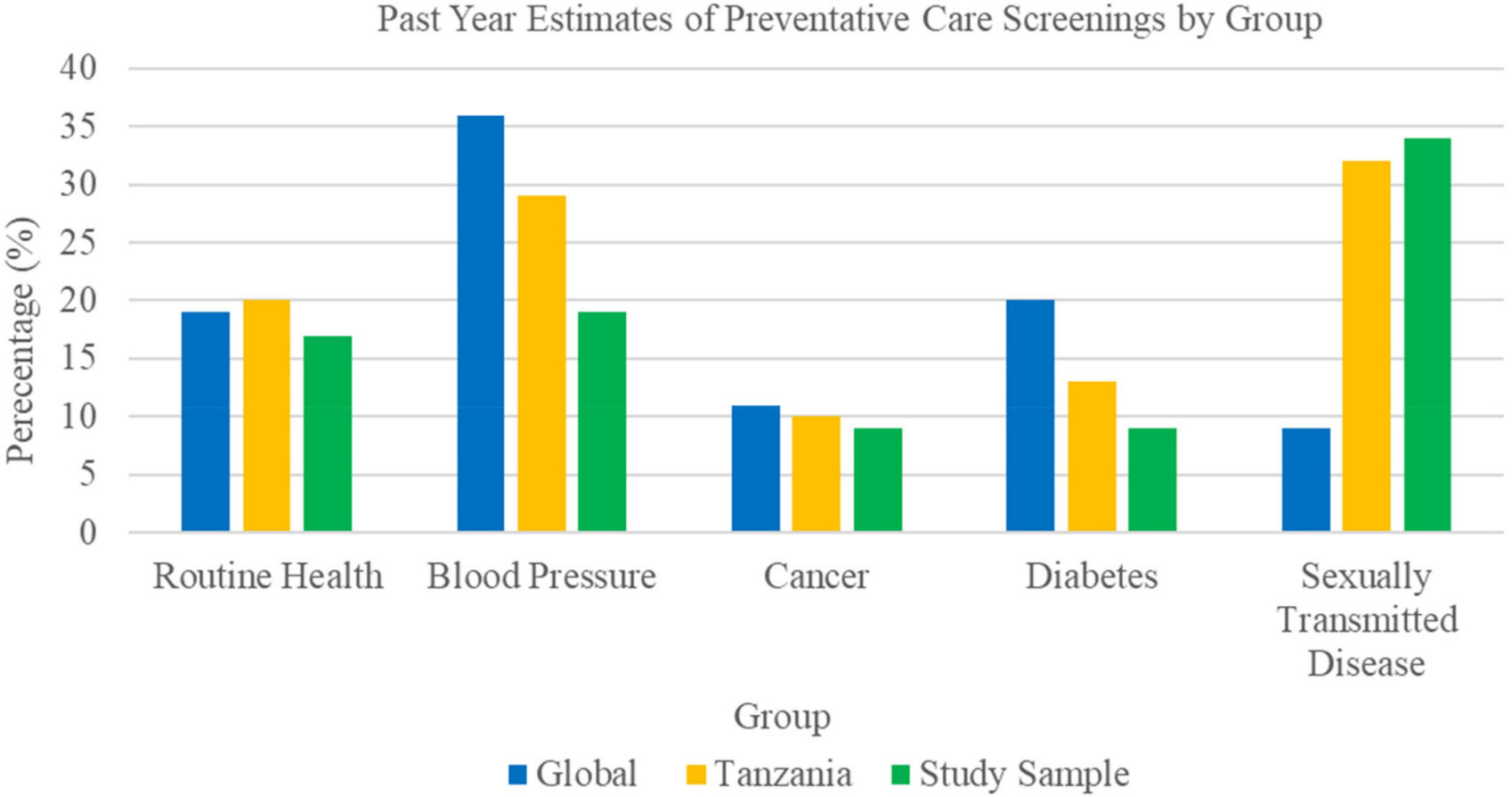
Comparative estimates of past year preventative care engagement*. *Data based on the 2024 three-year executive summary report from the Hologic Global Women's Health Index.

Among the 224 participants surveyed using the HGWI, past-year engagement with routine health, cancer, BP, and diabetes screenings was low and fell below both Tanzanian national and global benchmarks (see Figure 2). Only 18.8% (*n* = 42) of the sample reported having their BP checked, while just 9.4% (*n* = 21) had been screened for diabetes or cancer. In contrast, 34.4% (*n* = 77) had undergone sexually transmitted disease (STD) testing. Older participants (ages 51–75 and 76+) reported slightly higher past year engagement in routine health, BP and blood glucose screenings compared to younger adults (see Table 2).

**Table 2:** Percentage of past year health screenings reported in the study sample.

Past year health screenings		Health screenings by type					
		*n*	Routine health (%)	Blood pressure (%)	Cancer (%)	Diabetes (%)	Sexually transmitted disease (%)
	Total	224	17.97	18.75	9.38	9.38	34.38
Age	15–50	141	16.84	17.02	8.51	8.51	33.33
	51–75	71	19.72	22.54	9.86	9.86	36.62
	76+	12	20.83	16.67	16.67	16.67	33.33
Age at first pregnancy	No children	10	*0*	*0*	*0*	*0*	*0*
	< 18	104	8.65	2.88	*0*	*0*	31.73
	19–24	108	28.94	36.11	19.44	19.44	40.74
	25+	2	*0*	*0*	*0*	*0*	*0*
Income	Lowest 20%	43	19.19	18.6	13.95	13.95	30.23
	Second 20%	45	12.78	6.67	2.22	2.22	40.00
	Middle 20%	17	14.71	17.65	*0*	*0*	41.18
	Fourth 20%	38	22.37	28.95	15.79	15.79	28.95
	Highest 20%	29	24.14	24.14	17.24	17.24	37.93
	Unknown	52	15.87	19.23	5.77	5.77	32.69

Italicized values represent subgroup percentages within each demographic category (e.g., age group, age at first pregnancy, or income level).

## Discussion

This study provides novel insights into the intersection of oral health, NCDs, and women's health in a rural East African context. A pronounced pattern of clustered vulnerability emerged, with over 80% of participants meeting clinical thresholds for high oral disease burden. Within this group, a subset also presented with both elevated blood pressure and abnormal glucose levels, signaling an increased risk for adverse long-term health outcomes. Despite the application of appropriate statistical methods for each comparison, including both parametric and non-parametric approaches to address data distribution concerns, no statistically significant associations were identified between the examined variables and oral health outcomes in this study population.

However, high OHRQoL scores illustrate the significant functional and psychosocial burden associated with poor oral health. When considered alongside elevated neutrophil levels, these findings point to shared inflammatory pathways linking oral disease with systemic conditions such as cardiovascular and metabolic disorders. The convergence of these burdens, compounded by social disadvantage, reflects a syndemic pattern, wherein co-occurring conditions interact synergistically to worsen health outcomes.

Consistent with broader regional trends across sub-Saharan Africa, engagement with preventive care was notably low. Fewer than one in five participants had received a blood pressure screening in the past year, despite nearly half of those screened being hypertensive. One in five also exhibited abnormal glucose levels, most without a prior diagnosis, indicating possible undernutrition or undetected metabolic fragility. Participants without recent preventive care were more likely to report poor oral health, including higher DMFT scores, greater periodontal inflammation, and elevated systolic blood pressure.

Likewise, findings from the HGWI revealed persistent gaps in women's health, particularly in access to preventive services and routine screenings. Barriers to dental care, including limited-service availability and ongoing reliance on traditional oral hygiene practices, mirror structural challenges shaped by urbanization and constrained diagnostic infrastructure. These disparities can reflect entrenched inequities in access and underscore the continuing challenges of gender equity and empowerment in low-resource settings. Such gaps not only delay early detection and treatment but also perpetuate cycles of preventable disease and unmet health needs.

Although this study builds on prior qualitative research (22) using interviews and focus groups to explore women's perceptions of oral and systemic health, future studies should deepen and extend this foundation. Additional qualitative inquiry is needed to further illuminate the lived experiences, priorities, and health needs of women in similar settings. In-depth exploration of women's narratives can reveal how intersecting factors, such as cultural norms, caregiving responsibilities, stigma, financial constraints, and access to information, influence health-seeking behaviors and engagement with care. These insights are critical for designing interventions that are not only clinically effective but also culturally relevant and socially responsive, ultimately enhancing the equity and adaptability of community-based health systems in low-resource environments.

Collectively, results from the current study and similar research (22) highlight the urgent need for community-engaged, contextually grounded interventions that address both clinical and structural drivers of poor health. They also reinforce the value of integrated care models, such as the Common Risk Factor Approach, to address the overlapping biological, behavioral, and structural determinants of health. Integrating oral health with NCD prevention and women's health efforts, while remaining attuned to local cultural practices and resource limitations, will be essential moving forward. Such integrated strategies not only support more efficient use of scarce resources but also strengthen the capacity of local health systems to detect, manage, and prevent overlapping conditions. In due course, investing in holistic, equity-driven models of care offers a critical pathway to disrupt entrenched disadvantage and foster sustained health and well-being in underserved, resource-limited rural communities.

### Limitations

Although the study identified patterns suggesting a convergence of oral disease, inflammatory burden, and metabolic dysregulation among socially and educationally disadvantaged women, its findings are constrained by several limitations. The sample size (*n* = 224) was modest, and only a subsample (*n* = 45) underwent BP and glucose assessments, limiting statistical power and precluding multivariable analysis or control for confounders. As such, analyses were restricted to descriptive and bivariate comparisons, appropriate for the study's exploratory design. The cross-sectional nature of the study further limits causal inference, and the use of purposive, non-random sampling during community outreach introduces potential selection bias. Consequently, observed rates of untreated caries and undiagnosed hypertension may not reflect broader trends in the Rorya District or similar rural settings. Additionally, use of the PerioMonitor™ in rural field conditions presents challenges, as environmental variability such as lighting, humidity, and storage may affect test strip reliability and visual interpretation, underscoring the need for standardized protocols and field calibration. These limitations highlight the need for larger, representative studies to validate and expand upon these preliminary findings and to inform integrated, context-sensitive care strategies in resource-limited environments.

## Conclusion

Despite a high burden of untreated dental caries and periodontal inflammation, access to oral care remains limited in low-resource settings. This study demonstrates the feasibility and value of integrating biomarker-based diagnostics and multidimensional assessments into routine community health outreach. In particular, the novel application of PerioMonitor™ enabled early detection of subclinical inflammation, as evidenced by elevated neutrophil counts even among asymptomatic individuals. These results underscore the importance of early intervention strategies and highlight the urgent need for integrated, community-based approaches to oral health in rural Tanzania.

Findings from survey tools such as the OHRQoL and HGWI reveal that women experiencing poor health in one domain often face challenges in others, reflecting interconnected risks shaped by gender-related factors. The convergence of limited access to care, high rates of undiagnosed hypertension, abnormal glucose levels, and significant oral disease illustrates a syndemic pattern of clinical and structural vulnerability. These overlapping burdens emphasize the need for holistic, gender-sensitive care models that are responsive to the lived experiences of women in rural, low-resource settings.

While this research establishes that community-based screening in underserved settings is both feasible and impactful, it also lays the groundwork for developing scalable, sustainable models of care that integrate clinical services with meaningful community engagement. Expanding integrated screening approaches in low-resource contexts holds the potential to reduce health disparities, enable earlier detection of disease, and promote oral health equity. Future research efforts should prioritize larger, more representative samples to support multivariable and longitudinal analyses. At the same time, policy and programmatic initiatives should aim to decentralize diagnostic services and promote culturally grounded care that incorporates gender-responsive messaging.

To support integrated, sustainable care delivery, prevention strategies must embed routine oral and NCD screening, such as blood pressure, glucose, and periodontal inflammation assessments, into existing primary care and community health platforms. This could be achieved by training frontline health workers in basic screening protocols, equipping mobile clinics with low-cost diagnostic tools like the PerioMonitor™, and aligning screening efforts with maternal and child health services. NGOs and Ministries of Health can play a critical role by investing in workforce development, diagnostic infrastructure, and culturally tailored health education. Prioritizing such integration not only improves early detection and care coordination but also builds the foundation for resilient, equity-driven primary care systems in underserved settings.

## Data Availability

The raw data supporting the conclusions of this article will be made available by the authors, without undue reservation.

## References

[1] Beaglehole R Bonita R Horton R Adams C Alleyne G Asaria P Priority actions for the non-communicable disease crisis. Lancet. (2011) 377(9775):1438–47. 10.1016/s0140-6736(11)60393-0 21474174

[2] de-Graft Aikins A Unwin N Agyemang C Allotey P Campbell C Arhinful D. Tackling Africa’s chronic disease burden: from the local to the global. Global Health. (2010) 6:5. 10.1186/1744-8603-6-5 20403167 PMC2873934

[3] Mori AT. Mandatory health insurance for the informal sector in Tanzania—has it worked anywhere! perspective. Front Health Serv. (2023) 3. 10.3389/frhs.2023.1247301 37849823 PMC10577424

[4] Masalu JR Kikwilu EN Kahabuka FK Senkoro AR Kida IA. Oral health related behaviors among adult Tanzanians: a national pathfinder survey. BMC Oral Health. (2009) 9(1):22. 10.1186/1472-6831-9-22 19751519 PMC2749019

[5] Baelum V Fejerskov O Karring T. Oral hygiene, gingivitis and periodontal breakdown in adult Tanzanians. J Periodontal Res. (1986) 21(3):221–32. 10.1111/j.1600-0765.1986.tb01454.x 2941555

[6] Eid MA Selim HA al-Shammery AR. The relationship between chewing sticks (Miswak) and periodontal health. 3. Relationship to gingival recession. Quintessence Int. (1991) 22(1):61–4. 1784721

[7] Kikwilu EN Frencken JE Mulder J Masalu JR. Barriers to restorative care as perceived by dental patients attending government hospitals in Tanzania. Community Dent Oral Epidemiol. (2009) 37(1):35–44. 10.1111/j.1600-0528.2008.00446.x 19191819

[8] van Palenstein Helderman WH Lembariti BS van der Weijden GA van ‘t Hof MA. Gingival recession and its association with calculus in subjects deprived of prophylactic dental care. J Clin Periodontol. (1998) 25(2):106–11. 10.1111/j.1600-051x.1998.tb02416.x 9495609

[9] González-Olmo MJ Delgado-Ramos B Ortega-Martínez AR Romero-Maroto M Carrillo-Díaz M. Fear of COVID-19 in Madrid. Will patients avoid dental care? Int Dent J. (2022) 72(1):76–82. 10.1016/j.identj.2021.01.013 33743992 PMC7970159

[10] Peres MA Macpherson LMD Weyant RJ Daly B Venturelli R Mathur MR Oral diseases: a global public health challenge. Lancet. (2019) 394(10194):249–60. 10.1016/s0140-6736(19)31146-8 31327369

[11] Ataklte F Erqou S Kaptoge S Taye B Echouffo-Tcheugui JB Kengne AP. Burden of undiagnosed hypertension in Sub-Saharan Africa: a systematic review and meta-analysis. Hypertension. (2015) 65(2):291–8. 10.1161/hypertensionaha.114.04394 25385758

[12] Sato H Nakamura K Kibusi S Seino K Maro II Tashiro Y Patient trust and positive attitudes maximize non-communicable diseases management in rural Tanzania. Health Promot Int. (2023) 38(2):daad007. 10.1093/heapro/daad007 36884316

[13] Cresswell JA Alexander M Chong MYC Link HM Pejchinovska M Gazeley U Global and regional causes of maternal deaths 2009–20: a WHO systematic analysis. Lancet Glob Health. (2025) 13(4):e626–34. 10.1016/s2214-109x(24)00560-6 40064189 PMC11946934

[14] Bwana VM Rumisha SF Mremi IR Lyimo EP Mboera LEG. Patterns and causes of hospital maternal mortality in Tanzania: a 10-year retrospective analysis. PLoS One. (2019) 14(4):e0214807. 10.1371/journal.pone.0214807 30964909 PMC6456219

[15] Kibret GD Demant D Dawson A Hayen A. Spatial patterns of maternal and neonatal continuum of care use and its correlations with women’s empowerment. BMC Health Serv Res. (2024) 24(1):1018. 10.1186/s12913-024-11453-7 39227927 PMC11373502

[16] Glick M Monteiro da Silva O Seeberger GK Xu T Pucca G Williams DM FDI vision 2020: shaping the future of oral health. Int Dent J. (2012) 62(6):278–91. 10.1111/idj.12009 23252585 PMC9374976

[17] Glick M Williams DM Kleinman DV Vujicic M Watt RG Weyant RJ. A new definition for oral health developed by the FDI world dental federation opens the door to a universal definition of oral health. J Am Dent Assoc. (2016) 147(12):915–7. 10.1016/j.adaj.2016.10.001 27886668

[18] Glick M Williams DM Kleinman DV Vujicic M Watt RG Weyant RJ. A new definition for oral health developed by the FDI world dental federation opens the door to a universal definition of oral health. J Public Health Dent. (2017) 77(1):3–5. 10.1111/jphd.12213 28276588

[19] Glick M Williams DM Kleinman DV Vujicic M Watt RG Weyant RJ. A new definition for oral health developed by the FDI world dental federation opens the door to a universal definition of oral health. Br Dent J. (2016) 221(12):792–3. 10.1038/sj.bdj.2016.953 27981999

[20] Glick M Williams DM Kleinman DV Vujicic M Watt RG Weyant RJ. A new definition for oral health developed by the FDI world dental federation opens the door to a universal definition of oral health. Int Dent J. (2016) 66(6):322–4. 10.1111/idj.12294 27885673 PMC9376665

[21] Aminu K Jayasinghe RM Uwambaye P Salami A Murererehe J Ineza MC Oral health-related quality of life in the east African community: a scoping review. BMC Oral Health. (2025) 25(1):518. 10.1186/s12903-025-05921-7 40211243 PMC11987254

[22] Gudsoorkar P Nolan R Kafle S Dubey A. Exploration of oral hygiene practices, oral health status, and related quality of life of individuals residing in the Rorya district of Tanzania, east Africa. Front Oral Health. (2024) 5:1435555. 10.3389/froh.2024.1435555 39411580 PMC11473497

[23] Walsh T Worthington HV Glenny AM Marinho VC Jeroncic A. Fluoride toothpastes of different concentrations for preventing dental caries. Cochrane Database Syst Rev. (2019) 3(3):Cd007868. 10.1002/14651858.CD007868.pub3 30829399 PMC6398117

[24] Elebyary O Glogauer M Perio D. Monitoring oral inflammation: a chairside game-changer. Oral Health. (2023) 27(12):1–14. 10.1002/jper.11360

[25] Organization WH. Oral Health Surveys: Basic Methods. Geneva: World Health Organization (2013).

[26] Locker D Jokovic A Clarke M. Assessing the responsiveness of measures of oral health-related quality of life. Community Dent Oral Epidemiol. (2004) 32(1):10–8. 10.1111/j.1600-0528.2004.00114.x 14961835

[27] Schrouder S Agbodzakey JK Rhodd R Bore S. Addressing sustainable development goals in women’s health through collaborative governance: a Look at Africa. J Econ Sustain Dev. (2023) 14(12):102–10. 10.7176/JESD/14-12-11

[28] Glenny A-M Walsh T Iwasaki M Kateeb E Braga MM Riley P Development of tooth brushing recommendations through professional consensus. Int Dent J. (2023) 74(3):526. 10.1016/j.identj.2023.10.018 38052700 PMC11123540

[29] Dye BA. The global burden of oral disease: research and public health significance. J Dent Res. (2017) 96(4):361–3. 10.1177/0022034517693567 28318392 PMC6728669

[30] Ortíz-Barrios LB Granados-García V Cruz-Hervert P Moreno-Tamayo K Heredia-Ponce E Sánchez-García S. The impact of poor oral health on the oral health-related quality of life (OHRQoL) in older adults: the oral health status through a latent class analysis. BMC oral Health. (2019) 19(1):141. 10.1186/s12903-019-0840-3 31291933 PMC6622000

[31] Paul S. Using an American Heart Association Evidence-Based Protocol to Improve the Competency of Blood Pressure Measurement by Certified Nursing Assistants in a Geriatric Center. Vancouver, BC: Fairleigh Dickinson University (2019).

[32] Umpierrez GE Hellman R Korytkowski MT Kosiborod M Maynard GA Montori VM Management of hyperglycemia in hospitalized patients in non-critical care setting: an endocrine society clinical practice guideline. J Clin Endocrinol Metab. (2012) 97(1):16–38. 10.1210/jc.2011-2098 22223765

[33] Berk KN Carey P. Data Analysis with Microsoft Excel. Pacific Grove, CA: Duxbury Press (1998).

[34] Rossum G. Python Reference Manual. Amsterdam: CWI (Centre for Mathematics and Computer Science) (1995).

[35] Kinder JM Nelson P. A Student’s Guide to Python for Physical Modeling. Princeton, NJ: Princeton University Press (2021).

